# When Hyperglycemia Turns Black: Acute Necrotizing Esophagitis in a Catastrophic Metabolic Crisis: A Case Report

**DOI:** 10.3390/life16010134

**Published:** 2026-01-15

**Authors:** Corina-Ioana Anton, Roxana Lupu, Bogdan Mircea Petrescu, Cristian Sorin Sima

**Affiliations:** 1 Faculty of General Medicine, Carol Davila University of Medicine and Pharmacy, 8 Eroii Sanitari Bvd, 050474 Bucharest, Romania; corina-ioana.anton@drd.umfcd.ro (C.-I.A.); cristian.sima@drd.umfcd.ro (C.S.S.); 2 Department of Medico-Surgical and Prophylactic Disciplines, Titu Maiorescu University, 040441 Bucharest, Romania; 3 Department of Infectious Diseases, “Dr. Carol Davila” Central Military Emergency University Hospital, 134 Calea Plevnei, 010242 Bucharest, Romania; lupuroxana159@yahoo.com; 4 Department of Clinical Psychiatry, “Dr. Carol Davila” Central Military Emergency University Hospital, 134 Calea Plevnei, 010242 Bucharest, Romania; 5 Department of Urology, “Prof. Dr. Th. Burghele” Clinical Hospital, 20 Panduri Str., 050659 Bucharest, Romania

**Keywords:** acute necrotizing esophagitis, black esophagus, hyperglycemic crisis, diabetic ketoacidosis, thrombocytopenia

## Abstract

Background: Acute necrotizing esophagitis (ANE), also known as “black esophagus,” is a rare but life-threatening condition typically occurring in critically ill patients with profound systemic disturbances. Extreme hyperglycemic crises represent an underrecognized precipitating factor, capable of inducing severe metabolic, inflammatory, and microvascular injury. Case Presentation: We report the case of a 54-year-old male admitted with altered mental status and severe dehydration, in whom initial laboratory evaluation revealed extreme hyperglycemia (serum glucose ~1000 mg/dL), metabolic acidosis, and early multiorgan dysfunction. During intensive care unit hospitalization, the patient developed anemia and severe thrombocytopenia, followed by evidence of upper gastrointestinal bleeding. Urgent upper gastrointestinal endoscopy demonstrated diffuse circumferential black necrosis of the distal esophageal mucosa with abrupt demarcation at the gastroesophageal junction, consistent with acute necrotizing esophagitis, along with associated erosive hemorrhagic gastritis. Comprehensive laboratory evaluation documented marked inflammatory activation and hematologic instability. Management and Outcome: Treatment consisted of aggressive metabolic correction, strict glycemic control, hemodynamic stabilization, infection management, and supportive gastrointestinal care. Progressive clinical and biological improvement was observed, with resolution of bleeding and partial recovery of hematologic parameters. Conclusions: This case highlights a severe hyperglycemic crisis as a major contributing factor within a multifactorial ischemic and inflammatory cascade leading to acute necrotizing esophagitis.

## 1. Introduction

Acute necrotizing esophagitis (ANE), commonly referred to as black esophagus, is a rare but severe clinical entity characterized by circumferential black discoloration of the esophageal mucosa, predominantly involving the distal esophagus with abrupt demarcation at the gastroesophageal junction [1]. The reported incidence is below 0.3% in endoscopic series, yet mortality remains high, largely driven by underlying comorbidities and systemic insult rather than the esophageal lesion itself [2]. ANE is classically associated with states of profound hypoperfusion, metabolic derangements, sepsis, and diabetic emergencies [2].

Extreme hyperglycemic crises, particularly those exceeding conventional thresholds observed in diabetic ketoacidosis (DKA) or hyperosmolar hyperglycemic state (HHS), are infrequently reported as direct precipitants of ANE [3]. Severe hyperglycemia induces endothelial dysfunction, microvascular compromise, oxidative stress, and immune dysregulation, all of which synergistically impair mucosal defense and reparative mechanisms [3].

We report a uniquely severe case of acute necrotizing esophagitis occurring in the setting of an extreme hyperglycemic crisis (initial serum glucose ~1000 mg/dL) complicated by metabolic acidosis, sepsis, thrombocytopenia, and multiorgan dysfunction. This report aims to comprehensively document the clinical course, laboratory dynamics, endoscopic findings, and pathophysiological mechanisms linking catastrophic hyperglycemia to esophageal necrosis.

## 2. Case Presentation

A 54-year-old male with a known history of type 2 diabetes mellitus, chronic alcohol use disorder, and chronic pancreatitis was brought to the emergency department by ambulance after being found in a markedly altered general state. According to relatives, the patient had experienced several days of progressive asthenia, reduced oral intake, and mental confusion, followed by acute deterioration.

At presentation, the patient was severely dehydrated, pale, and poorly cooperative, with fluctuating levels of consciousness suggestive of a metabolic encephalopathy. Initial assessment revealed hemodynamic instability and signs of profound metabolic derangement, prompting immediate resuscitative measures. Despite the severity of presentation, there was no reported history of caustic ingestion, recent gastrointestinal instrumentation, or prior upper gastrointestinal bleeding.

Point-of-care testing disclosed an extreme hyperglycemic crisis, with an initial serum glucose level approaching 1000 mg/dL, accompanied by metabolic acidosis and acute renal dysfunction. This catastrophic metabolic state rapidly precipitated multiorgan involvement, necessitating admission to the intensive care unit for advanced monitoring and organ support.

The medical history was notable for long-standing heavy alcohol consumption, estimated by the patient at approximately 1 L of vodka per day, in addition to type 2 diabetes mellitus and chronic pancreatitis.

Upon physical examination after initial stabilization, the patient was conscious but intermittently disoriented. Blood pressure was approximately 121/57 mmHg, heart rate 63 bpm, and peripheral oxygen saturation 97–98% on room air. Cardiorespiratory examination revealed bilateral basal crackles, while abdominal examination showed a mildly distended but non-tender abdomen without signs of peritoneal irritation. No cutaneous stigmata of chronic liver disease or active bleeding were noted.

The early hospital course was dominated by the need for aggressive metabolic correction, including continuous intravenous insulin infusion, fluid resuscitation, electrolyte replacement, and broad-spectrum antimicrobial therapy initiated for suspected bacterial pneumonia. Laboratory investigations revealed a rapidly evolving systemic inflammatory response, progressive anemia, and severe thrombocytopenia.

During intensive care hospitalization, the patient developed evidence of upper gastrointestinal bleeding manifested by anemia progression and hemodynamic lability. Given the critical clinical context and absence of alternative explanations, urgent upper gastrointestinal endoscopy was performed. The examination revealed extensive circumferential necrosis of the distal esophageal mucosa with characteristic black discoloration and a sharp transition at the gastroesophageal junction, findings diagnostic of acute necrotizing esophagitis. Associated erosive and hemorrhagic gastritis was also observed.

A surgical consultation was obtained during hospitalization. In the absence of esophageal perforation, mediastinitis, or uncontrolled hemorrhage, a conservative management strategy was jointly agreed upon, with surgical intervention reserved for the development of complications.

Following diagnosis, management remained primarily supportive, focusing on sustained hemodynamic stabilization, strict glycemic control, acid suppression, nutritional optimization, and continued treatment of the underlying infectious and metabolic triggers. Gradual clinical improvement was observed in parallel with correction of metabolic abnormalities and inflammatory markers.

## 3. Results

Section 3 summarizes objective clinical, laboratory, and endoscopic data collected during hospitalization. All numerical values are transcribed exactly as documented in the provided medical records and are presented without interpretation beyond factual description.

### 3.1. Glycemic and Metabolic Parameters

Arterial blood gas analysis performed at presentation demonstrated profound metabolic acidosis, with a pH of 6.96, markedly reduced bicarbonate (5 mmol/L), and a strongly negative base excess (−15 mmol/L), accompanied by elevated lactate levels (12 mmol/L), consistent with severe metabolic derangement (Table 1). Respiratory compensation was evident, with a low PaCO_2_ of 15 mmHg, while oxygenation remained relatively preserved. This demonstrates mixed high-anion-gap metabolic acidosis (ketoacidosis and lactic acidosis). The patient exhibited hemodynamic compromise (MAP 58 mmHg, requiring vasopressors) and oliguria (urine output 300 mL in the first 24 h). Serum sodium was mildly low, likely reflecting hyperglycemia-induced pseudohyponatremia, and calculated osmolality indicated a hyperosmolar state. Urine and serum ketone measurements confirmed severe ketoacidosis.

#### Corrected Sodium and Hyperosmolar State

At presentation, measured serum sodium was 130 mmol/L, consistent with hyperglycemia-related pseudohyponatremia. After correction for severe hyperglycemia (serum glucose ~1000 mg/dL), the estimated corrected sodium was approximately 144 mmol/L, indicating a significant free water deficit and marked hyperosmolar dehydration. This finding supports substantial intravascular volume depletion and impaired tissue perfusion at admission.

Table 2 summarizes the glycemic profile and basic renal function parameters recorded during hospitalization. At presentation, the patient exhibited extreme hyperglycemia, with an initial serum glucose value of approximately 1000 mg/dL. Subsequent measurements documented marked glycemic variability, including hypoglycemia (58 mg/dL on 22 September 2025) and persistent hyperglycemia (396 mg/dL on 24 September 2025). Serum urea and creatinine values ranged between 17–19 mg/dL and 0.53–0.57 mg/dL, respectively.

### 3.2. Inflammatory and Infectious Markers

Table 3 presents inflammatory and infection-related biomarkers. C-reactive protein values were markedly elevated, with a peak of 147.01 mg/L on 20 September 2025 and a subsequent decrease to 81.41 mg/L on 22 September 2025. Procalcitonin was measured at 0.73 ng/mL, and ferritin concentration was 403.7 µg/L.

### 3.3. Hematologic Parameters

Table 4 summarizes erythrocyte and leukocyte parameters. Hemoglobin values ranged from a minimum of 8.2 g/dL to 9.0 g/dL at later assessment. Hematocrit values ranged between 23.7% and 25.5%. Leukocyte counts reached a minimum of 3.75 × 10^3^/µL during hospitalization. Red blood cell indices, including mean corpuscular volume and red cell distribution width, are detailed in the table.

### 3.4. Platelet Parameters

Table 5 details platelet counts and indices. The platelet count reached a nadir of 36 × 10^3^/µL and subsequently increased to 113 × 10^3^/µL by 24 September 2025. Mean platelet volume ranged from 12.3 to 13.1 fL. Plateletcrit and the proportion of large platelets are also reported.

### 3.5. Coagulation Profile

Table 6 summarizes coagulation parameters. Fibrinogen values ranged between 609 and 678 mg/dL. International normalized ratio values ranged from 1.14 to 1.20, while activated partial thromboplastin time values ranged from 27.4 to 28.1 s.

### 3.6. Evaluation of Thrombocytopenia and Microangiopathic Processes

The laboratory findings argue against thrombotic thrombocytopenic purpura, hemolytic uremic syndrome, or overt disseminated intravascular coagulation. Thrombocytopenia was therefore interpreted as multifactorial, predominantly driven by sepsis-associated platelet consumption, systemic inflammation, and critical illness (Table 7).

Severe thrombocytopenia raised consideration of heparin-induced thrombocytopenia (HIT), which can present with platelet consumption, thrombosis, and end-organ ischemia. In the present case, HIT was considered unlikely due to the absence of prior heparin exposure before platelet decline and the early onset of thrombocytopenia at presentation.

Application of the 4T score yielded a low pretest probability for HIT (thrombocytopenia present but without appropriate timing, thrombosis, or alternative heparin exposure), and therefore further immunologic testing was not pursued. This approach is consistent with current recommendations, which discourage laboratory testing for HIT in patients with a low 4T score due to poor positive predictive value.

#### Evaluation of Thrombocytopenia

Severe thrombocytopenia prompted a structured diagnostic evaluation. Peripheral blood smear showed only rare schistocytes (1–2/HPF) without evidence of significant microangiopathic hemolysis. Lactate dehydrogenase was only mildly elevated, haptoglobin was preserved, and no biochemical evidence of hemolysis was present, arguing against thrombotic thrombocytopenic purpura or hemolytic uremic syndrome. Coagulation parameters, including INR, APTT, and fibrinogen, were not consistent with overt disseminated intravascular coagulation.

Heparin-induced thrombocytopenia was considered unlikely due to absence of prior heparin exposure before platelet decline and a low 4T score. No clinical features suggested marrow failure or drug-induced thrombocytopenia. The overall pattern was most consistent with sepsis-associated platelet consumption and critical illness-related thrombocytopenia.

Given the profound thrombocytopenia and friable necrotic mucosa, endoscopic biopsy was deliberately deferred to minimize perforation and bleeding risk.

### 3.7. Hepatic and Enzymatic Parameters

Table 8 presents liver function tests and enzymatic markers. Alanine aminotransferase values ranged from 25 to 30 U/L, aspartate aminotransferase from 29 to 38 U/L, and gamma-glutamyl transferase from 56 to 65 U/L. Total protein concentration was 4.97 g/dL. Total and direct bilirubin values remained within documented ranges.

### 3.8. Endoscopic Findings

Table 9 summarizes the upper gastrointestinal endoscopic findings. The distal third of the esophagus was circumferentially involved by diffuse black necrotic mucosa, with an abrupt demarcation at the gastroesophageal junction. The mucosa was friable, with contact bleeding, but no active hemorrhage or perforation was observed. Associated erosive and hemorrhagic gastritis was documented, while the duodenum appeared macroscopically normal.

**Figure 1:**
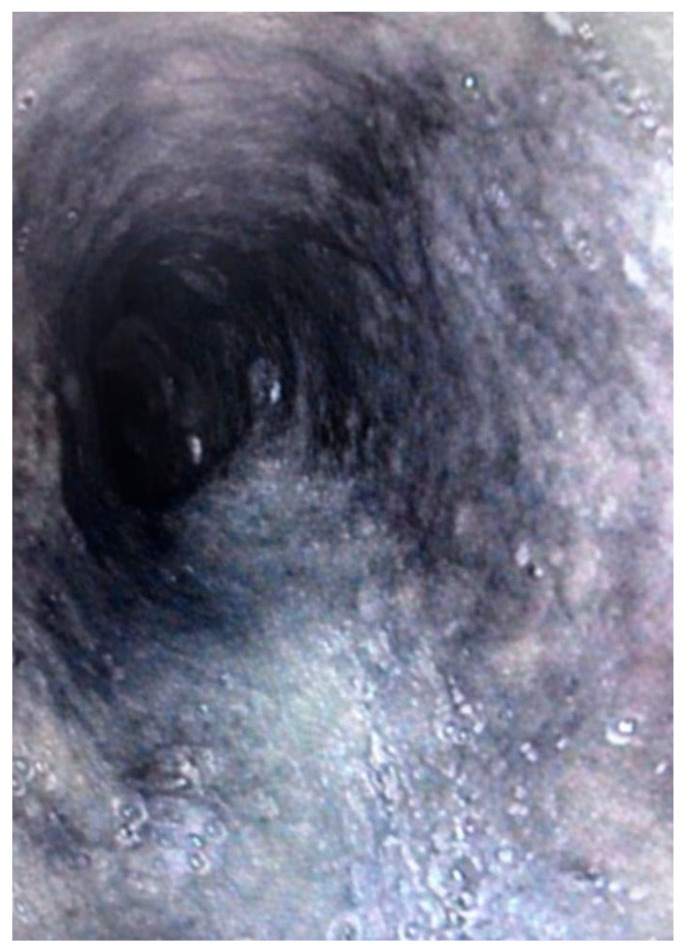
Endoscopic appearance of acute necrotizing esophagitis. Upper gastrointestinal endoscopy showing diffuse circumferential black discoloration of the distal esophageal mucosa, consistent with acute necrotizing esophagitis. (“black esophagus”). The mucosa appears friable, edematous, and necrotic, with loss of normal vascular pattern. These findings are characteristic of ischemic–necrotic injury of the esophagus occurring in the setting of severe systemic illness.

Upper gastrointestinal endoscopy revealed diffuse circumferential black discoloration of the distal esophageal mucosa, with marked friability and loss of normal mucosal architecture, findings diagnostic of acute necrotizing esophagitis (Figure 1).

## 4. Discussion

Acute necrotizing esophagitis (ANE) is widely recognized as a rare but dramatic manifestation of critical systemic illness rather than an isolated gastrointestinal disorder [3]. The present case illustrates a particularly severe form of ANE developing in the context of an extreme hyperglycemic crisis, with an initial serum glucose level approaching 1000 mg/dL, accompanied by marked inflammatory activation, hematologic instability, and multiorgan dysfunction. The coherence between metabolic, laboratory, and endoscopic findings strongly supports a multifactorial ischemic–metabolic pathogenesis.

### 4.1. Pathophysiological Links Between Extreme Hyperglycemia and Esophageal Necrosis

Acute necrotizing esophagitis is increasingly understood as the final manifestation of a multifactorial ischemic–inflammatory cascade rather than a primary esophageal disorder [4]. Extreme hyperglycemia can act as a powerful precipitating factor through several converging mechanisms that synergistically impair esophageal mucosal perfusion, cellular resilience, and reparative capacity [4].

Severe hyperglycemia induces marked hyperosmolarity, leading to osmotic diuresis, intravascular volume depletion, and reduced effective circulating volume [5]. The resulting hypovolemia compromises splanchnic perfusion and lowers regional oxygen delivery, particularly in the distal esophagus, which has limited vascular redundancy and is especially vulnerable to ischemic injury [4,5]. Concomitant metabolic acidosis and elevated lactate further impair mitochondrial oxidative metabolism, aggravating tissue hypoxia and cellular energy failure.

At the microvascular level, hyperglycemia promotes endothelial dysfunction through impaired nitric oxide bioavailability, increased oxidative stress, and enhanced expression of adhesion molecules [5]. These changes increase vascular resistance, promote capillary flow heterogeneity, and impair autoregulatory vasodilation, leading to regional malperfusion despite apparently preserved systemic blood pressure [4]. Hyperglycemia also increases blood viscosity and erythrocyte rigidity, further compromising microcirculatory flow [4,5].

Oxidative stress and non-enzymatic glycation of proteins weaken epithelial barrier integrity and blunt mucosal defense mechanisms [6]. In parallel, hyperglycemia induces immune dysregulation and amplifies systemic inflammatory activation, with cytokine-mediated endothelial injury, increased vascular permeability, and delayed epithelial regeneration [5,6]. These mechanisms converge to produce a fragile mucosal environment highly susceptible to ischemic necrosis under conditions of even transient hypoperfusion [6].

In the present case, these metabolic effects were compounded by shock physiology, severe lactic acidosis, and systemic inflammatory activation, creating a synergistic ischemic–inflammatory milieu capable of precipitating transmucosal esophageal necrosis. The profound acidemia observed at presentation reflects extreme systemic metabolic stress and impaired tissue perfusion, both of which are recognized contributors to ischemic gastrointestinal injury.

#### Comparison with Previously Reported Hyperglycemic ANE Cases

Published cases of ANE associated with diabetic ketoacidosis or hyperosmolar hyperglycemic state typically report glucose values in the range of approximately 400–800 mg/dL and frequently involve dehydration and variable degrees of hypotension [7,8]. Most reported patients recover with conservative management once metabolic derangements are corrected. However, few reports describe glucose levels approaching the extreme magnitude observed in the present patient (~1000 mg/dL), and detailed hematologic or inflammatory profiles are often limited [8,9].

The exceptional degree of hyperglycemia in this case may represent a threshold beyond which microvascular autoregulatory mechanisms fail, leading to critical regional ischemia despite early resuscitation. The coexistence of severe metabolic acidosis, elevated lactate, systemic inflammation, anemia, and thrombocytopenia likely further impaired oxygen delivery and microvascular integrity, amplifying mucosal vulnerability. This constellation suggests that not only the presence but also the magnitude and duration of hyperglycemia, combined with systemic inflammatory burden, may influence the severity of esophageal injury and clinical outcome.

Collectively, this case supports a model in which extreme hyperglycemia initiates dehydration, endothelial dysfunction, oxidative stress, and inflammatory amplification, which converge with shock and metabolic failure to produce catastrophic ischemic injury of the esophageal mucosa.

### 4.2. Systemic Inflammatory Response and Infectious Burden

The inflammatory profile documented in this case is notable for markedly elevated C-reactive protein levels, with a peak exceeding 140 mg/L, alongside increased procalcitonin and ferritin concentrations. These findings indicate a robust systemic inflammatory response, consistent with severe infection and metabolic stress [10]. Inflammation plays a central role in the pathogenesis of ANE by exacerbating endothelial dysfunction, increasing vascular permeability, and impairing mucosal repair mechanisms [10].

Hyperferritinemia further supports the presence of an intense acute-phase response and may reflect macrophage activation in the context of systemic illness. The coexistence of bacterial pneumonia likely amplified cytokine-mediated injury, compounding ischemic damage to the esophageal mucosa.

#### Evaluation of Infectious Etiology and Role of Sepsis

Acute necrotizing esophagitis is frequently reported in association with systemic infection, septic shock, and prolonged hypotension [10]; therefore, careful evaluation for infectious etiologies was an essential component of this case. At presentation, the patient demonstrated a marked systemic inflammatory response, with significantly elevated C-reactive protein, moderately increased procalcitonin, and hyperferritinemia, suggesting an acute inflammatory or infectious process. Clinical examination and imaging findings were consistent with suspected bacterial pneumonia, and empiric broad-spectrum antimicrobial therapy was initiated promptly.

Serial monitoring of inflammatory biomarkers demonstrated a downward trend in C-reactive protein following initiation of antimicrobial therapy and metabolic stabilization, supporting adequate control of the suspected infectious focus. Hemodynamic parameters improved with fluid resuscitation and vasopressor support without evidence of persistent refractory shock. No clinical signs of bloodstream infection, endocarditis, intra-abdominal sepsis, or invasive fungal disease were identified during hospitalization. Blood cultures and routine microbiological testing did not reveal persistent bacteremia or an alternative infectious source.

Importantly, the temporal relationship between the extreme metabolic derangement and the development of AEN suggests that hyperglycemic crisis and circulatory compromise were primary drivers of mucosal ischemia, with infection acting as a contributing but not exclusive factor. Severe dehydration, hyperosmolarity, endothelial dysfunction, and shock physiology were already present at admission, preceding the onset of gastrointestinal bleeding and endoscopic diagnosis of ANE.

While occult infection cannot be entirely excluded in critically ill patients, the absence of progressive septic deterioration, improving inflammatory markers, and favorable clinical response to supportive therapy argue against uncontrolled sepsis as the sole precipitating mechanism. Instead, this case supports a multifactorial model in which extreme hyperglycemia synergizes with systemic inflammation to precipitate microvascular ischemic injury of the esophagus.

### 4.3. Hematologic Abnormalities and Oxygen Delivery Impairment

The hematologic findings demonstrate significant anemia and leukopenia, with hemoglobin levels reaching a nadir of 8.2 g/dL and leukocyte counts falling below the lower reference limit. Reduced hemoglobin concentration directly compromises oxygen delivery to tissues already exposed to hypoperfusion, thereby lowering the threshold for ischemic necrosis [11].

Red blood cell indices remained within the normocytic range, suggesting an acute process rather than a chronic hematologic disorder. The temporal association between anemia progression and gastrointestinal bleeding further supports a clinically significant mucosal injury [12].

### 4.4. Thrombocytopenia and Microvascular Dysfunction

Severe thrombocytopenia was a prominent feature of this case, with platelet counts reaching a nadir of 36 × 10^3^/µL and only partial recovery during hospitalization. The concomitant increase in mean platelet volume and proportion of large platelets suggests peripheral platelet consumption with compensatory marrow response.

Thrombocytopenia is particularly relevant in ANE, as it contributes to mucosal bleeding risk and impairs reparative processes [13]. Moreover, platelet dysfunction in the setting of systemic inflammation may exacerbate microvascular thrombosis, further impairing esophageal perfusion despite relatively preserved global coagulation parameters [13].

### 4.5. Coagulation Profile and Acute-Phase Reactivity

Despite severe thrombocytopenia, coagulation times remained largely within reference ranges. However, fibrinogen levels were markedly elevated, reflecting acute-phase reactivity. Elevated fibrinogen increases blood viscosity and promotes a prothrombotic microenvironment, which may contribute to microcirculatory compromise without overt disseminated intravascular coagulation.

This dissociation between platelet count and coagulation times highlights the complexity of hemostatic alterations in critical illness and underscores the importance of evaluating coagulation status beyond standard clotting times alone [14].

#### Anticoagulation Considerations

The role of anticoagulation in ANE is limited and must be individualized. Unlike HIT or thrombotic microangiopathies, ANE is primarily driven by hypoperfusion, metabolic stress, and inflammatory microvascular dysfunction rather than large-vessel thrombosis.

In the present case, anticoagulation was not initiated due to severe thrombocytopenia, active gastrointestinal bleeding risk, and the absence of documented thromboembolic events. Supportive management aimed at correcting hemodynamic instability, metabolic derangements, and infection was prioritized.

Anticoagulation may be indicated only when a specific prothrombotic condition is identified, such as confirmed HIT, venous thromboembolism, or disseminated intravascular coagulation with a thrombotic phenotype, none of which were present in this patient.

### 4.6. Hepatic and Protein Metabolism Alterations

Liver enzyme levels remained within or near normal ranges, suggesting the absence of primary hepatocellular injury. However, total protein levels were reduced, indicating impaired nutritional status, acute-phase redistribution, or dilutional effects. Hypoproteinemia is a recognized contributor to impaired mucosal defense and delayed tissue repair, further predisposing to necrotic injury in the gastrointestinal tract [15].

### 4.7. Endoscopic Correlation and Diagnostic Significance

Endoscopic evaluation revealed classic features of acute necrotizing esophagitis, including circumferential black discoloration of the distal esophagus with an abrupt demarcation at the gastroesophageal junction. The absence of perforation and active hemorrhage at the time of endoscopy, despite marked mucosal friability, underscores the importance of timely diagnosis before catastrophic complications occur.

The coexistence of erosive hemorrhagic gastritis supports a shared ischemic–inflammatory mechanism affecting the upper gastrointestinal tract. The sparing of the duodenum is consistent with the segmental vascular vulnerability of the esophagus and stomach [16,17].

Chronic heavy alcohol consumption may have further impaired mucosal defense mechanisms and nutritional status, potentially lowering the ischemic threshold of the esophageal mucosa in this patient.

### 4.8. ANE as a Marker of Multisystem Organ Failure

Taken together, the findings in this case support the concept that ANE represents an end-organ manifestation of multisystem organ failure, rather than a primary esophageal disease. Extreme hyperglycemia, systemic inflammation, hematologic instability, and microvascular dysfunction converged to produce a critical ischemic insult to the esophageal mucosa [18].

The gradual improvement observed following aggressive metabolic correction, infection control, and supportive ICU management reinforces the principle that treatment of ANE must focus on reversal of the underlying systemic derangements rather than local intervention alone.

Despite oliguria and elevated urea at presentation, serum creatinine remained within low–normal ranges, likely reflecting reduced muscle mass, dilutional effects, and early aggressive fluid resuscitation, suggesting transient functional renal impairment rather than established intrinsic acute kidney injury.

### 4.9. Multidisciplinary Management and Surgical Considerations

Acute necrotizing esophagitis represents a complex clinical entity that frequently necessitates a multidisciplinary approach involving internists, intensivists, gastroenterologists, and surgeons. Although the management of ANE is predominantly conservative during the acute phase, early surgical consultation is strongly recommended, particularly in patients with extensive circumferential necrosis, severe comorbidities, or ongoing systemic instability.

Endoscopic evaluation, while essential for diagnosis, carries a non-negligible risk of esophageal perforation in the setting of profound mucosal ischemia, where the esophageal wall may become extremely fragile. For this reason, procedural decisions should ideally be made within a multidisciplinary framework, balancing diagnostic benefit against procedural risk.

In the present case, the absence of perforation or transmural necrosis allowed for successful conservative management. Nevertheless, surgical expertise remains crucial both for early recognition of complications and for planning potential downstream interventions.

Recent advances in minimally invasive and robotic-assisted esophageal surgery have expanded reconstructive options for complex benign esophageal disease. Robotic approaches may offer enhanced precision, reduced surgical trauma, and improved postoperative recovery when performed in specialized centers, particularly in patients requiring delayed reconstruction following ischemic or necrotic esophageal injury [19].

#### Therapeutic Strategy and Rationale

Continuous intravenous insulin infusion was initiated to achieve controlled reduction in serum glucose, suppress ongoing ketogenesis, and correct metabolic acidosis, thereby improving endothelial function and microvascular perfusion. Aggressive isotonic fluid resuscitation was used to restore intravascular volume, correct hyperosmolar dehydration, and optimize splanchnic blood flow. Electrolyte abnormalities were closely monitored and corrected to prevent arrhythmias and further metabolic instability. This approach is consistent with established management principles for hyperglycemic crises and previously reported ANE cases associated with diabetic emergencies [7,8,9].

High-dose proton pump inhibitor therapy was administered to reduce gastric acid exposure, minimize secondary chemical injury to the ischemic esophageal mucosa, and facilitate epithelial healing. Acid suppression is widely recommended in ANE to limit ongoing mucosal damage and reduce the risk of bleeding and perforation. Enteral intake was initially withheld to minimize mechanical stress on the fragile esophageal wall and prevent aspiration during the acute phase [12,13]. Nutritional support was gradually reintroduced after clinical stabilization, with attention to protein repletion given the patient’s hypoproteinemia and chronic alcohol use, both of which impair mucosal repair.

Empiric broad-spectrum antibiotic therapy was initiated due to suspected bacterial pneumonia and elevated inflammatory markers. Although ANE itself does not mandate antibiotic treatment in the absence of infection, early antimicrobial coverage was justified in this case because systemic infection may exacerbate ischemic injury, endothelial dysfunction, and inflammatory activation. Antibiotic therapy was subsequently tailored based on clinical response and microbiological data.

Transfusion management was guided by hemoglobin trends, hemodynamic status, and evidence of gastrointestinal bleeding, with the goal of maintaining adequate oxygen delivery in the setting of impaired tissue perfusion. Severe thrombocytopenia increased bleeding risk and influenced procedural and pharmacologic decisions. Anticoagulation was deliberately avoided because of low thrombotic suspicion, absence of confirmed thromboembolic disease, active bleeding risk, and platelet counts below safe thresholds.

Surgical consultation was obtained early to assess for potential complications, including esophageal perforation, mediastinitis, or uncontrolled hemorrhage. In the absence of transmural necrosis or perforation on endoscopy and imaging, conservative management was favored, consistent with published recommendations. This multidisciplinary approach aligns with reported ANE management strategies emphasizing early systemic stabilization, cautious endoscopic evaluation, and reserve of surgical intervention for complications.

Overall, the favorable clinical trajectory observed in this patient reinforces that timely correction of metabolic derangements, aggressive supportive care, and careful monitoring for complications remain the cornerstone of ANE management.

### 4.10. Unique Clinical Insights and Potential Prognostic Implications

Although ANE remains a rare entity, cases associated with diabetic ketoacidosis or hyperosmolar hyperglycemic state have been increasingly reported. Most published reports describe patients with moderate to severe hyperglycemia, dehydration, and hypotension who recover with supportive management once metabolic derangements are corrected [7,8,9].

The present case provides several distinctive clinical insights. First, the magnitude of hyperglycemia approached extreme levels (~1000 mg/dL), exceeding those typically reported in prior cases. Such profound hyperglycemia likely reflects prolonged metabolic decompensation and may represent a threshold beyond which microvascular autoregulation becomes critically impaired, predisposing to severe ischemic mucosal injury.

Second, this patient exhibited severe thrombocytopenia at presentation, with platelet counts falling to 36 × 10^3^/µL. Thrombocytopenia is not commonly emphasized in published ANE case reports and may represent an underrecognized contributor to disease severity. Beyond bleeding risk, platelet dysfunction and consumption may impair endothelial integrity, microvascular hemostasis, and tissue repair, thereby amplifying ischemic injury at the mucosal level.

Third, marked systemic inflammatory activation, evidenced by elevated C-reactive protein, ferritin, and procalcitonin levels, suggests a high inflammatory burden that may synergize with metabolic stress and hypoperfusion to accelerate tissue necrosis. The coexistence of extreme hyperglycemia, thrombocytopenia, and systemic inflammation may therefore constitute a composite marker of disease severity and microvascular vulnerability in critically ill patients.

Recognition of this triad may assist clinicians in identifying patients at increased risk for gastrointestinal ischemic complications and may justify earlier endoscopic evaluation, closer hemodynamic monitoring, and aggressive supportive management. While causality cannot be definitively established from a single case, this observation generates a hypothesis that warrants further investigation in larger observational cohorts.

A comparison with representative published cases of hyperglycemia-associated acute necrotizing esophagitis is provided in Table 10 [4,7,8].

### 4.11. Alternative Etiologies Considered

There was no exposure to vasoconstrictive agents, nonsteroidal anti-inflammatory drugs, bisphosphonates, potassium chloride, or other medications known to cause pill esophagitis. No caustic ingestion was reported by family or emergency services, and no recent endoscopic or nasogastric instrumentation occurred prior to admission.

Given the absence of histologic confirmation, alternative etiologies were actively considered. There was no history of caustic ingestion, pill-induced injury, recent endoscopic procedures, or vasoconstrictive drug exposure prior to ICU admission.

The strengths of this report include detailed physiologic documentation of metabolic collapse, hemodynamic compromise, and endoscopic correlation. Limitations include the absence of histologic confirmation and the presence of multiple concomitant systemic insults, precluding definitive causal inference. Nevertheless, the temporal relationship between metabolic shock and esophageal necrosis supports a plausible pathophysiologic association.

The differential diagnosis of acute necrotizing esophagitis includes a range of ischemic, infectious, toxic, and hematologic conditions that may present with upper gastrointestinal bleeding and severe systemic illness. Caustic ingestion and pill-induced esophagitis were considered unlikely in this case due to the absence of a suggestive history and the characteristic endoscopic finding of an abrupt demarcation at the gastroesophageal junction, which favors ischemic injury.

Infectious esophagitis, including cytomegalovirus, herpes simplex virus, and fungal infections, was also considered; however, these entities typically present with focal ulcerations rather than diffuse circumferential black necrosis and occur predominantly in severely immunocompromised patients.

Vasculitic disorders and thrombotic microangiopathies may cause gastrointestinal ischemia but are usually associated with systemic features, laboratory evidence of hemolysis, or characteristic histologic findings, which were not present in this case.

Taken together, the clinical context of profound metabolic derangement, circulatory shock, and endoscopic appearance strongly supported ischemic necrosis secondary to critical illness rather than a primary esophageal pathology.

At present, there is no high-quality clinical evidence supporting the use of traditional or complementary medicine in the treatment of acute necrotizing esophagitis. The published literature on ANE consistently emphasizes supportive intensive care, correction of systemic derangements, acid suppression, and treatment of precipitating conditions as the cornerstone of management. In critically ill patients, the unpredictable pharmacologic effects, potential drug interactions, and safety concerns associated with unregulated herbal or alternative therapies further limit their applicability. Therefore, such approaches cannot be recommended outside of controlled research settings.

## 5. Conclusions

This case describes an association between extreme hyperglycemic crisis and the development of acute necrotizing esophagitis in the setting of concurrent systemic inflammation and hemodynamic compromise.

It highlights ANE as a severe and life-threatening manifestation of systemic metabolic collapse rather than an isolated gastrointestinal disorder. An extreme hyperglycemic crisis, with initial serum glucose levels approaching 1000 mg/dL, was the central precipitating event, occurring in the context of systemic inflammation, hematologic instability, and multisystem organ dysfunction.

The convergence of profound metabolic derangement marked inflammatory activation, anemia, severe thrombocytopenia, and microvascular dysfunction created a critical ischemic milieu culminating in esophageal necrosis and hemorrhagic gastric injury. Endoscopic findings provided decisive diagnostic confirmation and emphasized the importance of early evaluation in critically ill patients presenting with anemia or suspected upper gastrointestinal bleeding.

This report underscores the need for heightened clinical awareness of acute necrotizing esophagitis in patients with severe hyperglycemic crises, particularly when additional risk factors such as infection, malnutrition, and chronic alcohol abuse are present. Prompt recognition, aggressive metabolic correction, infection control, and supportive intensive care remain the cornerstone of management and may significantly influence clinical outcomes.

Given the coexistence of alcohol use disorder pancreatitis, causality cannot be established; rather, this observation is hypothesis-generating and supports further investigation into the contribution of severe metabolic derangement to ischemic esophageal injury rather than establishing causality.

## Figures and Tables

**Table 1:** Key Laboratory and Hemodynamic Parameters at Presentation.

Parameter	Value	Units	Clinical Comment
pH	6.96	—	Severe acidemia
PaCO_2_	15	mmHg	Respiratory compensation
HCO_3_^−^	5	mmol/L	Profound metabolic acidosis
Base excess	−15	mmol/L	Severe deficit
Lactate	8.0	mmol/L	Severe lactic acidosis
PaO_2_	95	mmHg	Acceptable oxygenation
SaO_2_	92	%	Mild desaturation
β-hydroxybutyrate	7.2	mmol/L	Severe ketoacidosis
Urine ketones	+++/++++	—	Strongly positive
Sodium	130	mmol/L	Pseudohyponatremia likely
Glucose	620	mg/dL	Severe hyperglycemia
BUN	48	mg/dL	Dehydration/AKI risk
Calculated osmolality	~310	mOsm/kg	Hyperosmolar state
MAP	58	mmHg	Hypotension
Vasopressor required	Yes	—	Norepinephrine
Urine output (first 24 h)	300	mL	Oliguria

Urine ketones were strongly positive (+++/++++), corresponding to high-grade ketonuria on semi-quantitative dipstick analysis. BUN: Blood urea nitrogen; MAP: mean arterial pressure; AKI: acute kidney injury.

**Table 2:** Glycemic and Renal Function Parameters.

Parameter	Value	Units	Reference Range
Serum glucose (initial, ER)	~1000	mg/dL	70–105
Serum glucose (22 September 2025)	58	mg/dL	74–106
Serum glucose (24 September 2025)	396	mg/dL	70–105
Urea	17–19	mg/dL	17–43
Creatinine	0.53–0.57	mg/dL	0.67–1.17

ER: Emergency room.

**Table 3:** Inflammatory Response and Infection-Related Parameters.

Parameter	Value	Reference Range
C-reactive protein (20 September 2025)	147.01 mg/L	0–5 mg/L
C-reactive protein (22 September 2025)	81.41 mg/L	0–5 mg/L
Procalcitonin	0.73 mg/L	0–0.5 mg/L
Ferritin	403.7 µg/L	21.81–274.66 µg/L

**Table 4:** Complete Blood Count and Erythrocyte Indices.

Parameter	Value	Reference Range
Leukocytes (24 September 2025)	3.75 × 10^3^/µL	4.1–10.9 × 10^3^/µL
Hemoglobin (22 September 2025)	8.2 g/dL	13.2–16.6 g/dL
Hemoglobin (24 September 2025)	9.0 g/dL	13.2–16.6 g/dL
Hematocrit	23.7–25.5%	40–52%
Erythrocytes	2.47–2.51 × 10^6^/µL	4.54–6.00 × 10^6^/µL
Mean corpuscular volume (MCV)	96.0 fL	86–100 fL
Mean corpuscular hemoglobin (MCH)	33.2 pg	26–32 pg
RDW-SD	45.6–46.5 fL	21.5–54.5 fL

RDW-SD: Red Cell Distribution Width-Standard Deviation.

**Table 5:** Temporal changes in platelet count and platelet indices.

Parameter	Value	Reference Range
Platelets (22 September 2025)	36 × 10^3^/µL	140–440 × 10^3^/µL
Platelets (24 September 2025)	113 × 10^3^/µL	140–440 × 10^3^/µL
Mean platelet volume (MPV)	12.3–13.1 fL	9.2–12.1 fL
Plateletcrit	0.04%	0.19–0.36%
Large platelets (>12 fL)	42.8–48.0%	–

**Table 6:** Coagulation Parameters.

Parameter	Value	Reference Range
Fibrinogen	609–678 mg/dL	276–471 mg/dL
INR	1.14–1.20	0.87–1.25
APTT	27.4–28.1 s	25.4–36.9 s
Quick time	12.5–13.1 s	9.4–12.5 s

INR: International Normalized Ratio; APTT: Activated Partial Thromboplastin Time.

**Table 7:** Laboratory evaluation for thrombotic microangiopathy in the setting of thrombocytopenia.

Parameter	Value	Interpretation
Peripheral smear	Mild schistocytes (1–2/HPF), no spherocytes	No overt TMA
LDH	420 U/L	Mild tissue injury
Haptoglobin	90 mg/dL	No significant hemolysis
D-dimer	2.5 µg/mL FEU	Inflammation/early consumption
HIT exposure	None	HIT excluded

HPF: High-power field (microscopic field at high magnification); TMA: Thrombotic microangiopathy; LDH: Lactate dehydrogenase; HIT: heparin-induced thrombocytopenia.

**Table 8:** Liver Function and Enzymatic Markers.

Parameter	Value	Reference Range
ALT	25–30 U/L	3–50 U/L
AST	29–38 U/L	3–50 U/L
Gamma-GT	56–65 U/L	5–55 U/L
Alkaline phosphatase	66–69 U/L	30–120 U/L
Total proteins	4.97 g/dL	6.6–8.3 g/dL
Total bilirubin	0.62 mg/dL	0.30–1.20 mg/dL
Direct bilirubin	0.15 mg/dL	0–0.20 mg/dL

ALT: Alanine aminotransferase; AST: Aspartate aminotransferase; Gamma-GT (GGT): Gamma-glutamyl transferase.

**Table 9:** Upper Gastrointestinal Endoscopic Findings.

Endoscopic Feature	Description
Esophageal segment involved	Distal third of the esophagus
Distribution of lesions	Circumferential, continuous involvement
Mucosal appearance	Diffuse black discoloration consistent with necrotic mucosa
Longitudinal extent	Approximately 10 cm of circumferential necrosis involving the distal esophagus
Distance from incisors (extent)	Necrotic mucosa extending from approximately 30 cm to 40 cm from the incisors
Z-line	Present, located at approximately 40 cm from the incisors
Gastroesophageal junction	Sharp and abrupt demarcation between necrotic esophageal mucosa and gastric mucosa
Mucosal friability	Markedly increased; contact bleeding observed
Active bleeding	No active arterial bleeding identified
Esophageal perforation	Not observed
Biopsy	Deferred due to high risk of bleeding and perforation
Gastric findings	Diffuse erythema with multiple hemorrhagic erosions (erosive hemorrhagic gastritis)
Duodenal findings	Macroscopically normal

**Table 10:** Comparison of Present Case and Representative Published Hyperglycemic ANE Cases.

Reference/Case	Peak Glucose (mg/dL)	Shock/Hypotension	Thrombocytopenia or Hematologic Abnormality	Outcome
Present Case	~1000 (extreme hyperglycemia)	Yes—initial shock physiology	Severe thrombocytopenia (present)	Recovered with supportive care
Iwamoto et al. [8], hyperosmolar syndrome	Not specified (hyperosmolar hyperglycemic state)	Not clearly reported	Not reported	Recovered with supportive therapy
Moss et al. [4], 63-year-old DKA case	severe DKA (exact glucose not always reported)	No overt shock documented	Not specifically reported	Improved; mucosa healed on repeat endoscopy
Kitawaki et al. [7], Gurvits syndrome (66-year-old)	730	Not documented as true shock	Normocytic anemia present	Recovered with conservative DKA/ANE management

DKA: Diabetic ketoacidosis; ANE: Acute necrotizing encephalopathy.

## Data Availability

The original contributions presented in the study are included in the article, further inquiries can be directed to the corresponding author.
